# A comprehensive review of histone modifications during mammalian oogenesis and early embryo development

**DOI:** 10.1007/s00418-025-02398-x

**Published:** 2025-06-28

**Authors:** Nazlican Bozdemir, Tuba Kablan, Efe Biyikli, Ozgur Cinar, Fatma Uysal

**Affiliations:** 1 Department of Histology and Embryology, Ankara Medipol University School of Medicine, 06050 Altindag, Ankara Turkey; 2 Department of Histology and Embryology, Ankara University School of Medicine, 06080 Altindag, Ankara Turkey

**Keywords:** Epigenetics, Histone modifications, Oogenesis, Early embryo development, Preimplantation embryos

## Abstract

The success of both oogenesis and early embryo development relies heavily on dynamic epigenetic regulation in which gene activity changes without affecting the underlying DNA sequence. Epigenetics works through two main mechanisms: DNA methylation and histone modifications. DNA methylation typically leads to gene silencing, while histone modifications can either activate or repress genes depending on the specific modification, histone type, and targeted amino acid residue. Histone modifications affect important DNA regulatory processes in which the histone core area as well as the N-terminal tails that extend from the core region are vulnerable to a variety of posttranslational modifications (PTMs), including methylation, citrullination (deimination), acetylation, phosphorylation, ubiquitination, SUMOylation, ribosylation, and lactylation. This review article focuses on what is known about changes in the histone modifications and how these modifications and their responsible enzymes operate throughout mammalian oocyte maturation and early embryo development, highlighting their crucial roles in these processes.

## Introduction

Epigenetic regulation consists of heritable variations in gene expression without alterations in the DNA sequence. The three main mechanisms of epigenetic regulations are histone modifications, DNA methylation, and RNA-based mechanisms (Allis and Jenuwein 2016). Histone modifications affect important DNA regulatory processes (transcription, replication, etc.) in which the histone core area as well as the N-terminal tails that extend from the core region are vulnerable to a variety of posttranslational modifications (PMTs), including methylation, citrullination (deimination), acetylation, phosphorylation, ubiquitination, SUMOylation, and ribosylation (Smith and Denu 2009b). Epigenetic regulation, particularly through histone modifications, plays a pivotal role in ensuring proper oocyte development and early embryogenesis, and its disruption may underlie some of the limitations and outcomes associated with assisted reproductive technologies (ART) (Canovas et al. 2017). In a study, it is revealed that vitrification had no additional effect on gene expression or histone modifications in in vitro produced blastocysts, as both vitrified and nonvitrified two-cell embryos showed increased *H19* and *MEST* expression, increased H3K9ac, and decreased H3K9me2 compared with in vivo controls (Jahangiri et al. 2014). Moreover, researchers found permissive histone mark H3K4me2 is significantly higher at the H19/IGF2 and KCNQ1OT1 differentially methylated regions (DMRs) in the placenta of in vitro fertilization (IVF)/intracytoplasmic sperm injection (ICSI) groups. In contrast, the repressive markers H3K9me2 and H3K9me3 were significantly reduced at the *H19/IGF2* and *SNURF* DMRs, indicating that ART-induced DNA hypomethylation at imprinted regions leads to a more permissive chromatin configuration (Choux et al. 2020). Furthermore, placental tissues from intracytoplasmic sperm injection (ICSI) exhibited global alterations in the H3K4me3 when compared with those from natural conceptions, whereas levels of H3K9ac and H3K27ac did not demonstrate significant change. Thus, ICSI boys had the highest number of genes with altered H3K4me3 in the cord blood mononuclear cell (CBMC) compared with the in vitro fertilization IVF group, suggesting sex- and method-specific epigenetic alterations (Yang et al. 2021a). In mouse IVF embryos, abnormal H3K4me3 modification at active promoters in the epiblast leads to increased expression of extraembryonic tissue-specific genes, including 334 epiblast-specific genes and 24 epiblast-specific transcription factors, in the extraembryonic ectoderm (ExE), and is considered to be a major cause of implantation failure and abnormal placental development (Bai et al. 2022). In our previous articles, we reviewed the DNA methylation (Uysal et al. 2015) and histone acetylation (Bozdemir and Uysal 2023) during mammalian oogenesis and early embryo development. In line with that, in this review article, we aimed to focus on what is known about alterations in the histone modifications, including histone methylation, phosphorylation, ubiquitination, SUMOylation, ribosylation, citrullination, and lactylation, and current advancements in the acetylation and how these modifications and their responsible enzymes operate throughout mammalian oocyte maturation and early embryogenesis.

## Methodology

A comprehensive literature search was conducted using PubMed to identify relevant publications on histone modifications during early embryogenesis and oogenesis. The search strategy incorporated key terms such as histone methylation, demethylation, acetylation, ubiquitination, phosphorylation, dephosphorylation, SUMOylation, ribosylation, lactylation, and citrullination, along with mammalian oogenesis and embryogenesis. To ensure relevance, we refined our search using both single-term and multiterm combinations and explicitly excluded data from nonmammalian studies. A literature search on acetylation was conducted for studies published between 2023 and 2025.

## Histone modifications

### Histone methylation

**Fig. 1:**
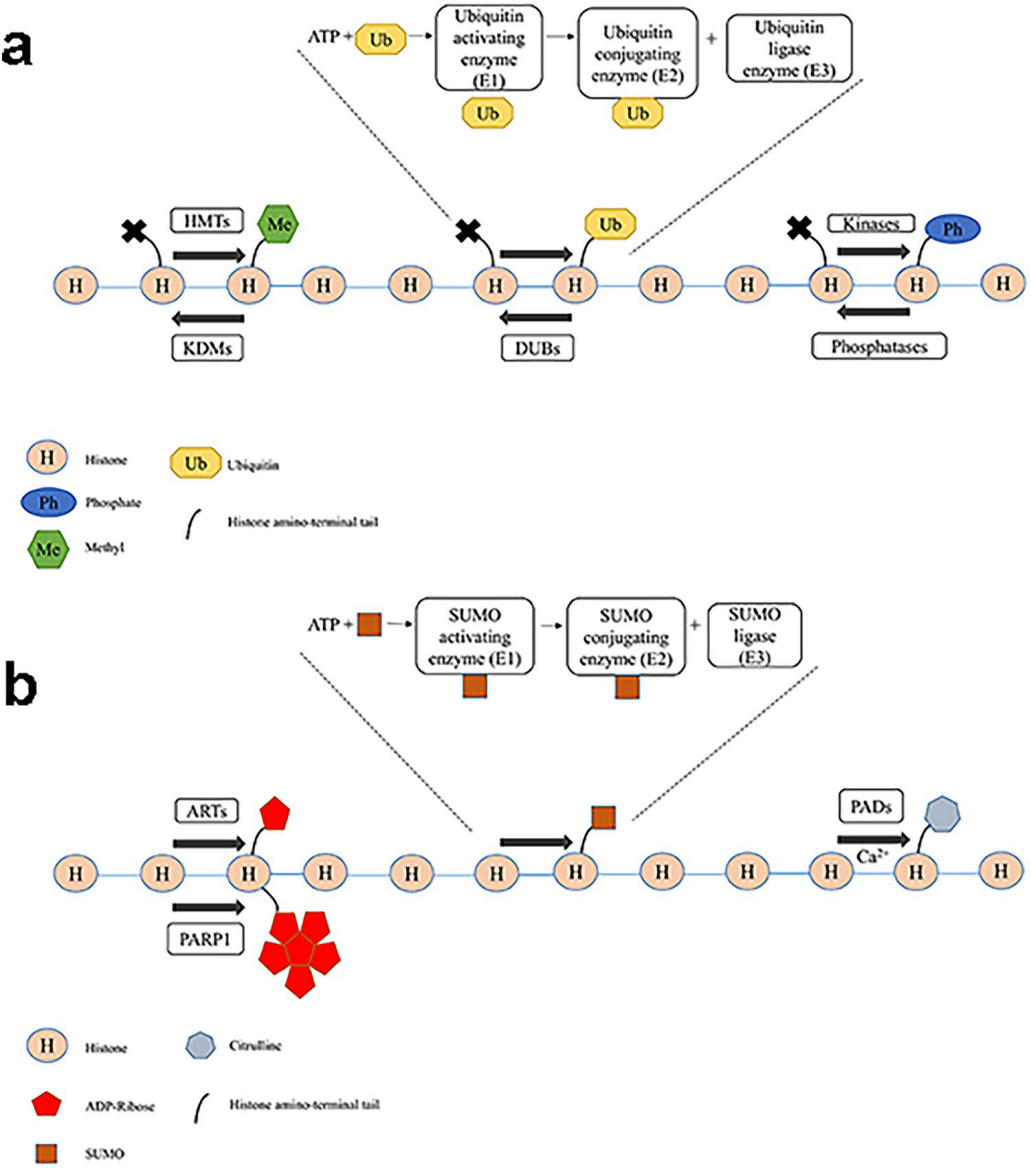
Representation of histone modifications. **a** Histone methylation, ubiquitination and phosphorylation. Histone methylation is established through histone methyltransferases (HMTs) and removed through histone demethylases (KDMs). Ubiquitination is established through E1, E2, and E3 ligase, while deubiquination is established through deubiquitinase (DUBs). Histone phosphorylation is maintained through kinases and phosphatases. **b** Histone ribosylation, SUMOylation, and citrullination. Histone ribosylation is maintained through ADP ribosyl transferase enzymes (ARTs) and polyribosylation is established through poly (ADP-ribose) polymerase I (PARP1). Histone SUMOylation is established through E1, E2, and E3 enzymes while histone citrullination is regulated by protein arginine deiminases (PADs)

The nucleosome, which is found in eukaryotic organisms, is the fundamental component of chromosomes. It is made up of double-stranded DNA wrapped around a protein octamer that contains two copies of each of the histone proteins H2A, H2B, H3, and H4 (Luger and Hansen 2005). Modifying certain amino acids in these proteins by adding methyl groups is a dynamic process called histone methylation (Fig. 1a) (Greer and Shi 2012). This mechanism facilitates or impedes transcription factors or other proteins to reach the DNA (Neidhart 2015). Moreover, the most frequent methylation mark acceptor sites within histone tails are basic amino acid chains of lysine (Lys or K), histidine (His or h), and arginine (Arg or R) positively charged amino acids (Smith and Denu 2009a; Strahl and Allis 2000; Kouzarides 2002) Thus, methyl groups do not alter the charge of the histone, in contrast to acetyl groups (Bannister and Kouzarides 2011). In addition, although histidines have only been discovered to be monomethylated, methyltransferases have been found to add several methyl groups to a single arginine or lysine, mono-, di-, or even tri-methylating the residue in the case of lysine and these mechanisms are catalyzed by protein arginine methyltransferases (PRMTs) and lysine methyltransferases (PKMTs) (Bannister and Kouzarides 2011; Greer and Shi 2012).

#### General roles of histone methyltransferases

Two distinct groups of PRMTs are responsible for catalyzing arginine methylation. Type I PRMTs including PRMT1, PRMT2, PRMT3, PRMT4, PRMT6, and PRMT8, and type II PRMTs including PRMT5 and PRMT9 are shown first (Bedford and Clarke 2009b). Moreover, it is known that PRMT7 is considered as type III PRMT (Feng et al. 2013). Asymmetric dimethylarginine (aDMA) is created by type I PRMTs attaching two methyl groups to a single terminal nitrogen atom. Besides, type II PRMTs catalyze the synthesis of symmetric dimethylarginine, which has a methyl group on each terminal nitrogen (Yang et al. 2015). Furthermore, the only type III PRMT known as PRMT7 solely generates monomethylated arginine (Feng et al. 2013). In addition, PRMTs need *S*-adenosyl-l-methionine (SAM) as a methyl-donating cofactor to methylate arginine. Moreover, the existence of arginine demethylases is uncertain (Yang and Bedford 2013), although prior research found that several lysine demethylases (KDM3A, KDM4E, and KDM5C) also had arginine demethylation activity in vitro (Walport et al. 2016).

PRMT1 is the primary enzyme that produces aDMA in proteins, and it prefers RGG/RG motifs (Thandapani et al. 2013) and methylates RNA-binding and DNA-damaging proteins to regulate RNA metabolism and maintain genome stability, respectively (Table 1) (Bedford and Clarke 2009b; Auclair and Richard 2013). It has been demonstrated that PRMT2 serves as a transcriptional repressor (Pak et al. 2011). The zinc finger at PRMT3’s N-terminus, which confers substrate specificity (Frankel and Clarke 2000) and methylates ribosomal proteins, sets it apart from other PRMTs (Swiercz et al. 2007). For its role as a transcription coactivator, PRMT4, also known as CARM1, is well-known. In addition to directly recruiting transcription factors and methylating H3R17 and H3R26, it also potentiates transcription (Suresh et al. 2021). By contrast, H3R2me2a is produced by PRMT6-mediated methylation, which is typically linked to transcriptional suppression (Neault et al. 2012)..

**Table 1:**
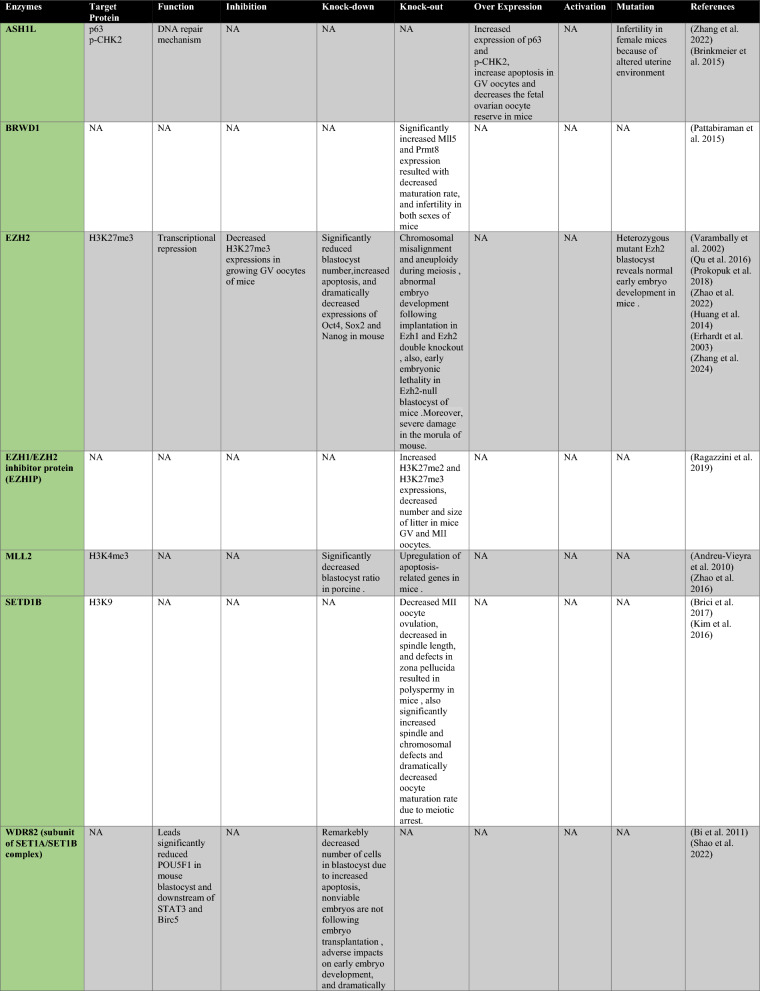

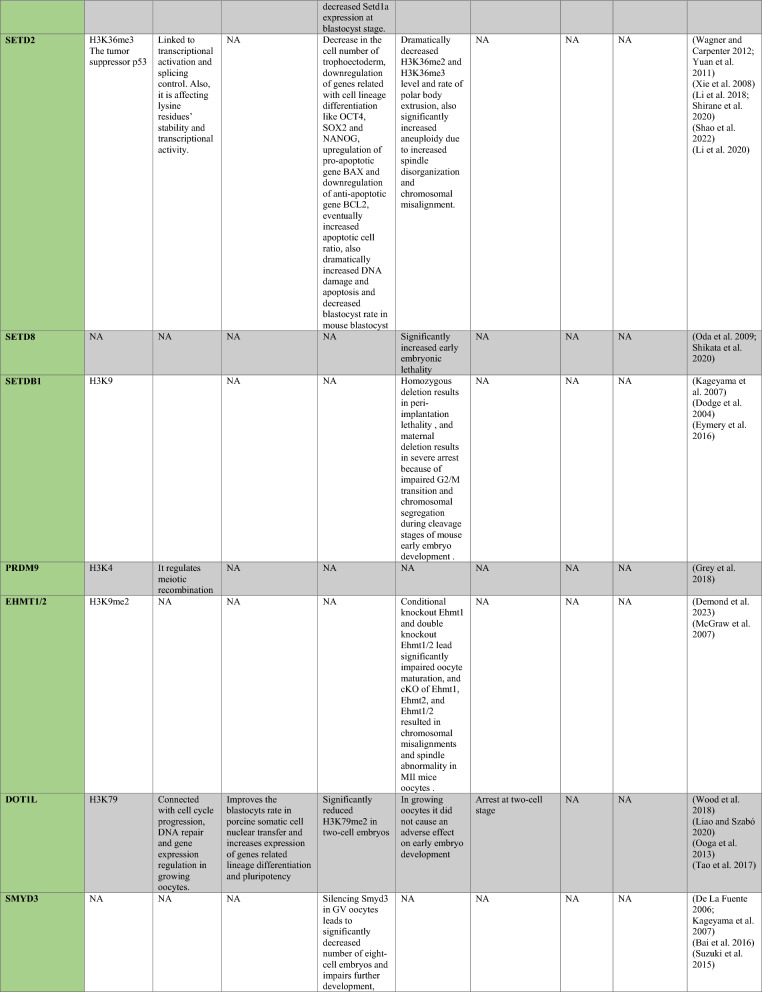

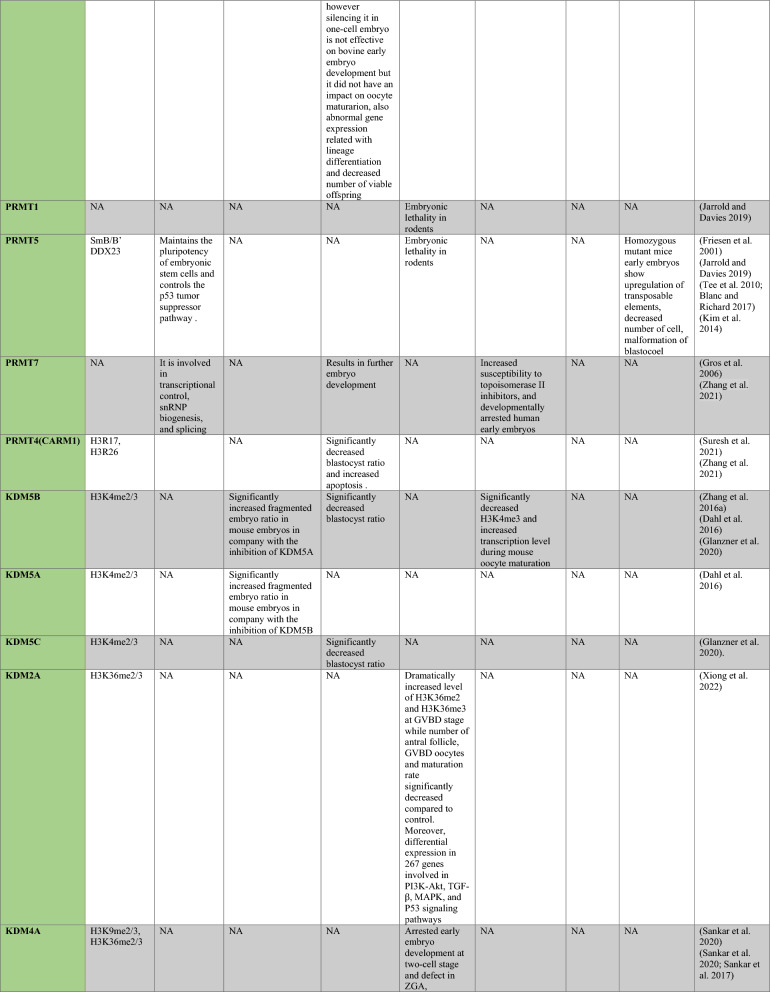

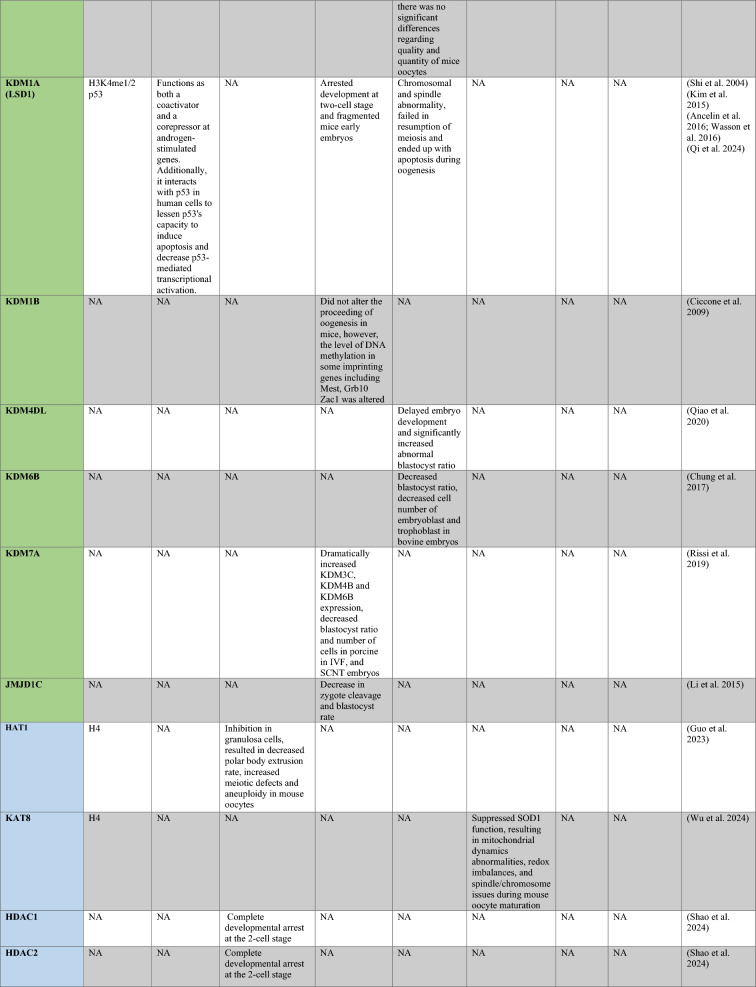

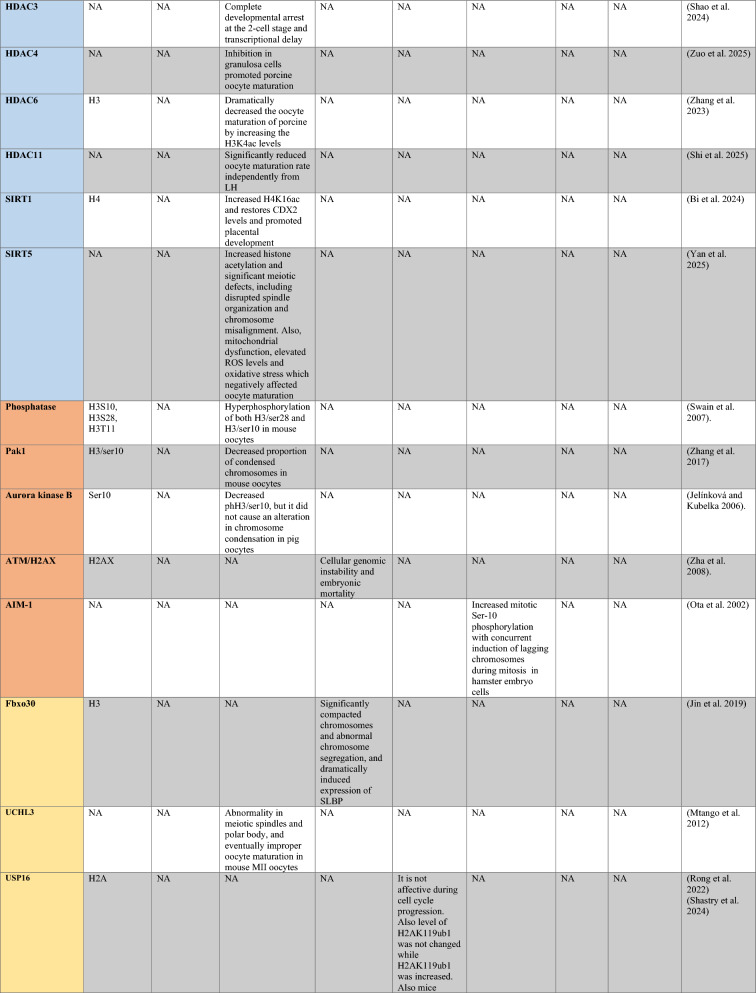

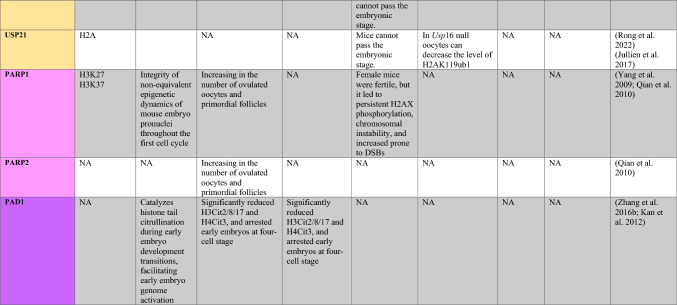
A summary table of histone modifying enzymes and their target proteins and roles in oogenesis and embryogenesis, as well as results from investigations including inhibition/activation, knockdown/overexpression, knockout, or mutation in mammalians

In the enzymes section, green stands for methyltransferases and demethylases, and blue stands for acetlytransferases and deactyltransferases. Orange stands for phosphatases and kinases; yellow stands for ubiquitinases and deubiquitinases, pink stands for ribosylation enzymes, and purple stands for citrullinases. *NA* not available

Asymmetric dimethylarginine (aDMA) and monomethylarginine (MMA) are formed on arginine residues by type II PRMTs. Type II PRMTs do not necessitate preexisting methylation on the target arginine residue, in contrast to type I PRMTs, which catalyze the symmetric dimethylarginine (sDMA). Instead, they catalyze aDMA or MMA by immediately transferring a methyl group from SAM to the guanidino nitrogen atom of the arginine residue. In addition, RNA processing, signal transmission, and chromatin remodeling are just a few of the cellular processes in which type II PRMTs have been shown to be crucial (Bedford and Clarke 2009a). For instance, it has been demonstrated that PRMT5, one of the most thoroughly investigated type II PRMTs, controls RNA splicing by methylating the splicing component small nuclear ribonucleoprotein associated proteins B and B′ (SmB/B′) and the RNA helicase DDX23 (Friesen et al. 2001). Additionally, PRMT5 has been linked to maintaining the pluripotency of embryonic stem cells as well as controlling the p53 tumor suppressor pathway (Tee et al. 2010; Blanc and Richard 2017). The type II enzyme PRMT9 has lately been demonstrated to not be redundant with PRMT5 since they catalyze the methylation of different substrates (Hadjikyriacou et al. 2015). Remarkably, PRMT9 activity can change from a type II to a type III enzyme when changes occur in the catalytic region (Jain et al. 2016). Moreover, except for the variants in PRMT9, PRMT7 is the only type III PRMT that is involved in transcriptional control, snRNP biogenesis, and splicing. The first screen for PRMT7 found that its depletion increased susceptibility to topoisomerase II inhibitors (Gros et al. 2006).

PKMTs are classified into subfamilies depending on structural characteristics and target proteins such as Su(var)3-9, enhancer-of-zeste, and trithorax (SET) domain-containing lysine methyltransferases (SET-domain PKMTs) and non-SET domain lysine methyltransferases (non-SET PKMTs) (Martin and Zhang 2005). The SET domain, a conserved catalytic domain identified in PKMTs with SET domains, is in charge of transferring methyl groups from SAM to lysine residues. SET-domain PKMTs, such as the enhancer-of-zeste 1 polycomb repressive complex 2 (EZH2), have been linked to transcriptional repression by trimethylation of lysine 27 on histone H3 (H3K27me3) (Varambally et al. 2002). SET-domain PKMTs can target nonhistone proteins in addition to histones, extending their functional versatility. For instance, the enzyme SETD2 is engaged in lysine 36 methylation on histone H3 (H3K36me), which is linked to transcriptional activation and splicing control (Wagner and Carpenter 2012; Yuan et al. 2011). SETD2 also methylates lysine residues on other nonhistone proteins, including the tumor suppressor p53, affecting its stability and transcriptional activity (Xie et al. 2008). Positive regulatory (PR) domain-containing protein 9 (PRDM9) is another SET-domain containing PKMT that is involved in the methylation of lysine 4 on histone H3 (H3K4). PRDM9 has been found to regulate meiotic recombination, which is essential for genetic diversity and fertility (Grey et al. 2018). Additionally, a study has found that embryos lacking G9a exhibit significantly reduced H3K9 methylation, leading to severe growth retardation and lethality. This suggests that euchromatic histone H3 lysine 9 methylation, regulated by the SET-domain methyltransferase G9a, is essential for early embryogenesis and plays a key role in the transcriptional repression of developmental genes (Tachibana et al. 2002). Non-SET domain lysine methyltransferases target both histone and nonhistone proteins, in contrast to their SET domain counterparts (Hyun et al. 2017). Disruptor of telomeric silencing 1-like (DOT1L), which methylates lysine 79 on histone H3 (H3K79), is an example of a non-SET PKMT, and its modification has been connected to the regulation of gene expression, DNA repair, and cell cycle progression (Wood et al. 2018).

#### Histone lysine demethylases (KDMs)

Depending on their sequence homologies and catalytic processes, KDMs are divided into two subfamilies. Members of the first subfamily to be discovered, the KDM1 subfamily (also known as lysine-specific demethylases, or LSDs) (Shi et al. 2004) is amine-oxidase domain-containing demethylases that use the cosubstrate flavin adenine dinucleotide (FAD) to catalyze demethylation and have been demonstrated to be crucial for animal growth, cellular differentiation, and gene expression (Hino et al. 2012) (Fig. 1a). Moreover, lysine-specific demethylase 1 (KDM1A or LSD1) removes one or two methyl groups from H3K4 (H3K4me1/2) with exceptional selectivity but cannot target trimethylated H3K4 (H3K4me3) (Shi et al. 2004). Furthermore, there is a proof that target genes’ transcription is repressed as a result of histone demethylation and deacetylation caused by the binding of histone deacetylases (HDACs) to LSD1 (Lee et al. 2006). The second subfamily consists of jumonji-domain-containing demethylases (JmjC domain-containing KDMs) (Trewick et al. 2005). About 20 human enzymes that are included in the JmjC domain-containing KDM class are the enzymes that catalyze the demethylation of mono-, di-, and tri-methylated lysines at various locations (Walport et al. 2012). Lysine demethylases with Jumanji domains play important roles during cellular differentiation and development by modifying the epigenetic landscape. (Cloos et al. 2006).

### Histone acetylation and deacetylation

Histone acetylation is an important epigenetic alteration that regulates gene expression through modulating chromatin configuration. This process is carried out by histone acetyltransferases (HATs), which transfer acetyl groups from acetyl-CoA to particular lysine residues on histone tails. The addition of acetyl groups neutralizes lysines’ positive charge, reducing the affinity between histones and the negatively charged DNA backbone. This relaxation of the chromatin structure provides access for transcription factors and the transcriptional machinery, therefore enhancing gene expression (Montgomery et al. 2015; He et al. 2021). HATs are divided into Types A and B based on their cellular localization (Fig. 3). In humans, the nucleus contains three Type A HATs families: GCN5-related *N*-acetyl transferases (GNAT) including hGCN5/KAT2A and PCAF/KAT2B, CREB-binding protein (CBP/KAT3A), and p300 (KAT3B), and the MYST family contains males absent on the first (MOF/KAT8/MYST1), monocytic leukemia zinc finger protein (MOZ/KAT6A/MYST3), MOZ-related factor (MORF/KAT6B/MYST4), HAT binding to the origin recognition complex 1 (HBO1/KAT7/MYST2), and HIV Tat interacting 60 kDa protein (TIP60/KAT5). Despite their different sequences, these enzymes have a structurally conserved acetyl-CoA binding core that is required for catalytic activity (McCullough and Marmorstein 2016). Histone deacetylases (HDACs), by contrast, catalyze histone deacetylation, which is related to gene suppression. There are four classes of HDACs in humans: the Class 1 enzymes (reduced potassium dependency (Rpd3-like) proteins) include HDAC1, HDAC2, HDAC3, and HDAC8; the Class II enzymes (Hda1-like proteins) include HDAC4, HDAC5, HDAC6, HDAC7, HDAC9, and HDAC10; the Class III enzymes (Sir2-like pro teins) include SIRT1, SIRT2, SIRT3, SIRT4, SIRT5, SIRT6, and SIRT7; and the Class IV enzyme includes HDAC11 (Seto and Yoshida 2014).

### Histone ubiquitination

Ubiquitination is an adenosine triphosphate (ATP)-dependent cascade process ligating ubiquitin and a type of the histone modification; however, it is different from the other histone modifications because it requires the covalent binding of a 76-amino acid protein. There are three enzymes establishing ubiquitination ,which are E1, an activating enzyme, E2, a conjugating enzyme, and E3, a ligase enzyme (Fig. 1a) (Sun et al. 2020; Mattiroli and Penengo 2021). E1 initially activates the C-terminal carboxyl group of ubiquitin before it forms a thioester bond with the enzyme’s catalytic cysteine. After that, ubiquitin is converted via a transesterification process to the catalytic cysteine of E2. The C-terminal glycine residue of ubiquitin is then transferred from E2 to the amino group of a substrate lysine residue by E3 ligase, which catalyzes the formation of an isopeptide bond between the lysine residue and ubiquitin residue (Sekiguchi and Matsushita 2022). According to the conjugate mechanism, there is monoubiquitination, multimonoubiquitination, and polyubiquitination. Monoubiquitination is the attachment of the single ubiquitin to a single lysine. Multimonoubiquitination is the attachment of the single ubiquitin to multiple lysine, and polyubiquitination implies that ubiquitin molecules are attached end-to-end to a lysine residue on a substrate protein to form a polyubiquitin chain (Sun et al. 2020). Therefore, ubiquitin molecules are conjugated with one of the seven lysine residues included on itself (K6, K11, K27, K29, K33, K48, and K63) or the first methionine (M1) (Sewduth et al. 2020).

Moreover, ubiquitin molecules can be removed by enzymes through a process called deubiquitination (Mevissen and Komander 2017). These enzymes are classified into two groups based on their mechanism: cysteine proteases and metalloproteinases (Park et al. 2020). They possess at least one ubiquitin-binding site, known as the S1 site, which facilitates enzyme–substrate interaction. Once bound to the S1 site, deubiquitination enzymes act on the ubiquitin molecules at the C-terminus, allowing the precise removal and processing of ubiquitin (Du et al. 2020).

### Histone phosphorylation and dephosphorylation

The process of adding a phosphate group to a molecule or an ion is known as phosphorylation (Zippo et al. 2009). The N-terminal tails of histones are the primary sites for phosphorylation; these tails can be phosphorylated by various kinases and dephosphorylated by phosphatases (Fig. 1a). All core histones include phosphoacceptor sites in their N-terminal domains, and it is a highly dynamic process that frequently occurs on the serine, threonine, and tyrosine residues of histone tails (Rossetto et al. 2012). The serine 10 (S10) residue on histone H3 is a key histone phosphorylation site that has been linked to gene activation (Wei et al. 1999). Furthermore, another research has shown that the catalytic acetyltransferase domain of general control nonderepressible 5 (Gcn5) phosphorylates S10 and accommodates the phosphate to enhance further acetylation at lysine-14 by Gcn5 (Lo et al. 2000). On histone H3, the serine 28 (S28) residue is another crucial phosphorylation site. The control of the cell cycle and the response to DNA damage are both affected by phosphorylation at this location. H3S28 phosphorylation contributes to genomic integrity by regulating how quickly DNA repair machinery may reach damaged regions (Dawson and Kouzarides 2012). Furthermore, during cell division, histone phosphorylation is dynamically controlled, as seen by the phosphorylation of H3-Ser10, H3-Thr11, and H3-Ser28 (Dong and Han 2012).

### Histone SUMOylation

Small ubiquitin-like modifier (SUMO) proteins are covalently linked to certain lysine residues on histone proteins during histone SUMOylation (Hendriks and Vertegaal 2016). A series of enzymatic events involving the SUMO-activating enzyme (E1), the SUMO-conjugating enzyme (E2), and the SUMO ligase (E3) drive the process. These enzymes work together to activate and transport SUMO proteins to targeted lysine residues, forming the SUMO-histone complex (Fig. 1b) (Song et al. 2004). Depending on the particular histone and target gene, SUMOylation has been linked to both gene activation and repression (Gill 2005; Chymkowitch et al. 2015). For instance, transcriptional suppression of genes involved in cell-cycle control has been associated with the histone H4 SUMOylation (Johnson 2004).

### Histone ribosylation

Adenosine diphosphate-ribosylation (ADPr) is a reversible posttranslational protein modification that regulates fundamental cellular and biological processes such as DNA damage repair, cell proliferation, and differentiation (Palazzo et al. 2019). ADPr is carried out by transferase enzymes (ARTs), which are categorized into two enzyme superfamilies on the basis of the similarity of their catalytic domain with bacterial toxins: the cholera toxin-like ADP-ribosyl transferases (ARTCs) and the diphtheria toxin-like ADP-ribosyl transferases (ARTDs) (Hottiger et al. 2010; Di Girolamo and Fabrizio 2019). ARTs covalently connect single or multiple ADP-ribose clusters from nicotinamide adenine dinucleotide (NAD+) to the substrates using NAD+ as the donor, resulting in mono ADP-ribosylation or poly ADP-ribosylation (PARylation) (Fig. 1b) (Hassa et al. 2006).

### Histone citrullination

Citrullination is a posttranslational alteration catalyzed by the peptidyl arginine deiminase (PAD) enzyme family, it depends on increasing calcium concentration (Zhu et al. 2021). PADs are a small class of enzymes that convert (Wang and Wang 2013) positively charged arginine residues to citrulline through a hydrolytic mechanism, resulting in histone citrullination and euchromatin structure (Klose and Zhang 2007) (Fig. 1b). In mammals, the family consists of five isotypes (PAD1–4 and PAD6), which are encoded by five distinct genes clustered in a single gene cluster on the p-arm of chromosome 1. Each PAD enzyme has a distinct subcellular localization, tissue distribution, and substrate specificity; however, it should be emphasized that no substrate proteins have yet been identified for PAD6 (Raijmakers et al. 2007). PAD6 has the highest expression levels in ovary and testis (Chavanas et al. 2004).Moreover, it has been reported that one of the organs where PAD1 is most expressed is the uterus.

### Histone lactylation

Histone lactylation is a newly discovered posttranslational modification that affects chromatin dynamics and gene expression by adding lactate molecules to lysine residues on histone proteins. Changes at histone H3 lysine 18 (H3K18la) and lysine 23 (H3K23la) are two major forms of histone lactylation in mammals. These modifications demonstrate dynamic changes during early development, and their levels peak at the blastocyst stage. In addition, their expressions are noticeable in fully mature germinal vesicle (GV) oocytes (Lu et al. 2024). The production of lactate by cellular metabolism, particularly glycolysis, is strongly linked to the formation of histone lactylation (Dai et al. 2022). Lactate links cellular metabolism to epigenetic regulation by serving as a substrate for protein lactylation. Since they produce lactate, enzymes such as lactate dehydrogenase A (LDHA) are essential to this process; inhibition of LDHA has been demonstrated to lower histone lactylation levels and impair mouse embryonic development (Yang et al. 2021b). Thus, the histone acetyltransferase p300 is one of the major lactylation catalyzers. It has been demonstrated that it can also catalyze the transfer of lactyl groups to lysine residues on histones such as H3K18 and H4K5 by utilizing lactyl-CoA as a donor (Zhang et al. 2019). More recently, it was shown that alanyl-tRNA synthetases, specifically AARS1 and AARS2, are new lactyltransferases that efficiently transfer lactate to lysine residues and promote protein lactylation by producing high-energy intermediates such as lactyl-AMP (Sahu and Lu 2025). By contrast, members of the HDAC family are histone lactylation erasers. HDAC1, HDAC2, and HDAC3 have been shown in vitro to efficiently remove lactyl groups from histones, namely from H3K18la and H4K5la, reversing lactylation and promoting chromatin remodeling (Zessin et al. 2022). Furthermore, delactylase activity is demonstrated by the sirtuin family (SIRT1-7) of NAD⁺-dependent deacetylases, including SIRT1, SIRT2, and SIRT3. It has been demonstrated that SIRT2 operates in the cytoplasm, whereas SIRT3 functions in the mitochondria, indicating compartment-specific control over protein lactylation (Liu et al. 2024)

## Histone modifications during oogenesis

Primordial germ cells (PGCs) are derived from pluripotent epiblast cells and function as gamete progenitors. PGCs migrate from the yolk sac endoderm to the bilateral genital ridges during development. PGCs multiply mitotically throughout migration and, eventually differentiate into spermatogonia or oogonia depending on the sex chromosome (Geijsen et al. 2004). Oogenesis is the process through which oocytes mature. Oogonia enter meiotic division to create primary oocytes during oogenesis and arrest at prophase I. These meiotically arrested oocytes in prophase I are known as germinal vesicle (GV) oocytes, and they remain in the ovary until puberty. However, when puberty approaches, the nuclear envelope dissolves to allow meiosis to restart, and these oocytes are referred to as GV breakdown (GVBD) oocytes. GVBD oocytes continue meiosis and develop through the metaphase I (MI) and metaphase II (MII) phases. However, oocytes are halted in the MII stage once more, and they can only complete meiosis if fertilized by a sperm (Sánchez and Smitz 2012; Filatov et al. 2019). Oocyte development and maturation are dependent on the timing of gene expression. As a result, epigenetic changes and enzymes involved in epigenome control are extremely important (Bozdemir and Uysal 2023).

### Histone methylation

**Fig. 2:**
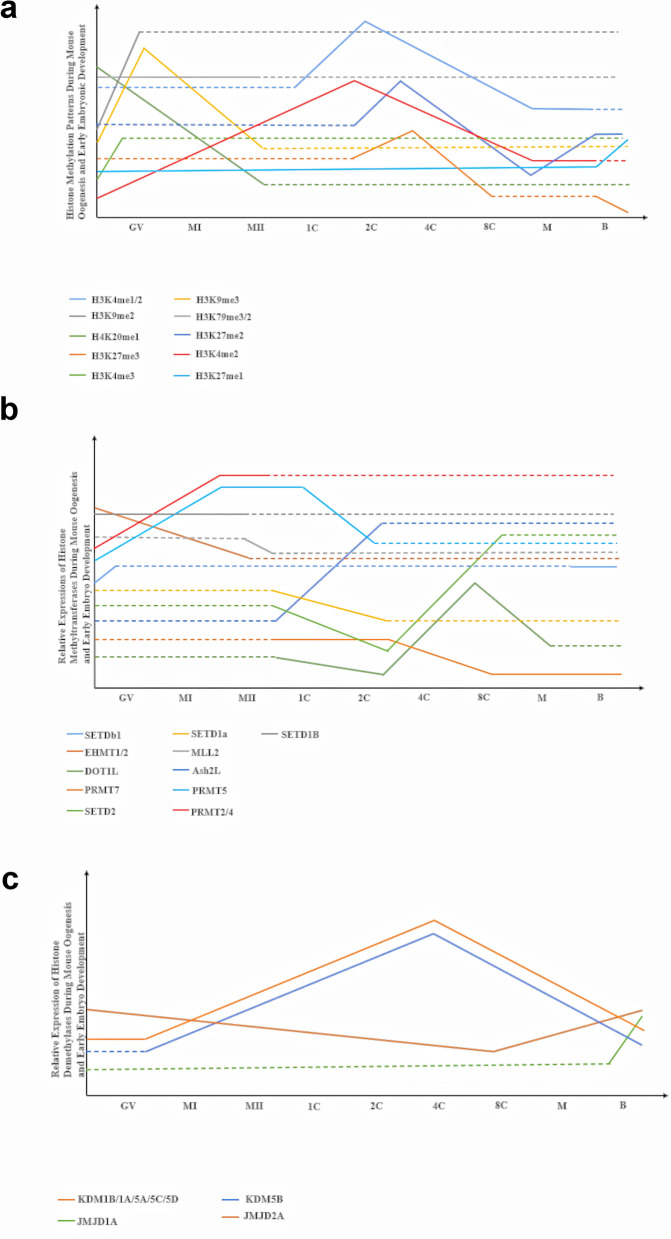
Dynamic pattern of histone methylation, histone methyltransferases, and demethylases during mouse oogenesis and early embryo development. **a** Relative level of histone methylation throughout oogenesis and early embryo development. **b** Relative expression of histone methytransferases throughout oogenesis and early embryo development. **c** Relative expression of histone demethylases throughout oogenesis and early embryo development. Dashed line indicates not available. *GV* germinal vesicle, *MI* metaphase I, *MII* metaphase II, *1C* one-cell stage, *2C* two-cell stage, *4C* four-cell stage, *8C* eight-cell stage, *M* morula stage, *B* blastocyst

H3K4me2 and H3K4me3 are associated with euchromatin structure, and mammalian oocytes exhibited increasing H3K4me2 and H3K4me3 until GV stage; H3K4me2 and H3K4me3 staining was not seen in heterochromatin configuration at this stage. The expression of SET7, which is responsible for H3K4me2 but not for H3K4me3, increased until GV stage. SET, myeloid-Nervy-DEAF-1 (MYND)-containing domain 3 (SMYD3), and mixed lineage leukemia (MLL) revealed fluctuating expression patterns during oocyte growth, increasing in the fully grown GV oocyte; it is uncertain whether these enzymes are responsible for the increased H3K4me3 at GV stage (De La Fuente 2006; Kageyama et al. 2007). By contrast, H3K9me2 and H3K9me3 are associated with increased transcriptional repression at the GV stage of mice oocytes. Suppressor of variegation 3–9 homolog (SUV39H or KMT1A), ERG-associated protein with SET domain (ESET or SETDB1), G9a (EHMT2), and G9a-like protein (GLP) are responsible for methylation of H3K9, and their expression increased until the GV stage (Kageyama et al. 2007). SETD2 knockout and silencing in mice oocytes led to dramatically decreased H3K36me2 and H3K36me3 levels, decreased rates of polar body extrusion, and increased spindle disorganization and chromosomal misalignment, eventually resulting in significantly increased aneuploidy (Li et al. 2018; Shirane et al. 2020). H3K79me2 and H3K79me3 were found from GV to MII stages of mice oocytes; however, they disappeared immediately following fertilization (Ooga et al. 2008). In mice oocytes, H4K20me1 level significantly decreased from the GV stage to MII stage (Luo et al. 2013). H3R17me and H4R3me were present throughout the nucleus at the GV stage of mice, but only faintly colocalized with the chromatin. After meiotic maturation, methylation of H3R17 and H4R3 were removed from the chromosomes of MII stage oocytes (Hatanaka et al. 2017). By contrast, research on human oocytes revealed that H4R3 maintained a steady methylation status from the GV to MII stage (Gu et al. 2010). In ob/ob mice GV oocytes, H3K27me2 level significantly decreased while H3K9me2 level increased compared with the control group, and a high-fat diet did not have a dramatic impact on both H3K27me2 and H3K9me2 level compared with the control group (Fig. 2a) (Hou et al. 2016).

#### Histone methyltransferases

It has been shown that the overexpression of absent, small, or homeotic-like 1 (ASH1L) resulted in increased expression of p63 and p-checkpoint kinase 2 (p-CHK2) which are necessary for DNA repair mechanism; in turn, this eventually led to increased apoptosis in GV oocytes and decreased fetal ovarian oocyte reserves in mice (Zhang et al. 2022). The absence of bromodomain and WD repeat domain containing 1 (BRWD1) led to infertility in both sexes of mouse, and it was revealed that in GV oocytes of the *Brwd1* knockout mouse, Mll5 and Prmt8 expression were significantly increased and resulted in a dramatically decreased maturation rate due to increased chromosome instability compared with wild type (Pattabiraman et al. 2015). It was shown that Mll expression increased throughout oocyte maturation from GV to MII oocytes, and *EZH2* knockout in mouse oocytes caused chromosomal misalignment and aneuploidy during meiosis (Qu et al. 2016). Additionally, inhibition of EZH2 with tazemetostat, which is used in cancer treatment, results in decreased H3K27me3 in growing mouse GV oocytes (Prokopuk et al. 2018). By contrast, knockout of cellular EZH1/EZH2 inhibitor protein (*EZHIP*) in mouse GV and MII oocytes resulted in increased H3K27me2 and H3K27me3 and showed decreased number of litter and litter size (Ragazzini et al. 2019). Moreover, in a recent study, they investigated the role of EZH1/2 in the methylation of H3K27 in mouse oocyte meiosis by inhibiting its activity and deleting its gene and found cell apoptosis and a reduction in oocyte numbers within embryonic ovaries, demonstrating that it is required for oocyte development during meiosis prophase I in mice (Jiang et al. 2024). In addition, maternal *Ezh1/2* deletion altered the expression of mitochondrial-related genes in MII oocytes (Zhang et al. 2024).

MLL2 is responsible for H3K4me3 and its expression is increased in GV oocytes; however, conditional *Mll2*-knockout mice revealed significantly decreased ovulation rate, H3K4me2 and H3K4me3 level, and upregulation of apoptosis-related genes (Andreu-Vieyra et al. 2010). Additionally, it is known that H3K4me3 and DNA methylation are antagonists and that the presence of H3K4me3 prevents the genomic region from undergoing DNA methylation. However, in conditional *Mll2* knockout, DNA methylation was mostly similar to GV oocytes of Mll2 wild-type mouse (Hanna et al. 2018). Moreover, *Setd1b* conditional knockout (cKO) mouse oocytes demonstrated that decreased MII oocyte ovulation, decreased in spindle length, and defects in zona pellucida and resulted in polyspermy (Brici et al. 2017). Furthermore, *SMYD3* mRNA expression was found to be similar throughout oogenesis; however, downregulation of *SMYD3* did not have an impact on oocyte maturation rate (Bai et al. 2016). Additionally, EHMT1 and EHMT2 are responsible for H3K9me2. *EHMT1* mRNA expression is increased, while *EHMT2* expression is not altered during bovine oocyte maturation (McGraw et al. 2007). In addition, conditional-knockout *Ehmt1* and double-knockout *Ehmt1/2* significantly impaired oocyte maturation, and cKO of *Ehmt1*, *Ehmt2*, and *Ehmt1/2* resulted in chromosomal misalignments and spindle abnormality in mouse MII oocytes (Demond et al. 2023). SUV39H1 and SUV39H2 are responsible for H3K9me3 in mouse oocytes. It was found that H3K9me3 was significantly decreased in MII oocytes compared with GV oocytes in bovine models, and, consistently, *SUV39H1* and *SUV39H2* mRNA expression decreased from GV stage to MII stage oocytes (Zhang et al. 2016b). SETD1B catalyzes the dimethylation of H3K9; the number of abnormal oocytes, spindle, and chromosomal defects significantly increased, and oocyte maturation rate dramatically decreased due to meiotic arrest in *Setd1b*-knockout mice (Kim et al. 2016). *PRMT2* and *PRMT5* expressions were dramatically decreased during bovine oocyte maturation (McGraw et al. 2007). Additionally, the deletion of *Dot1l* gene in growing oocytes did not cause an adverse effect on early embryo development; therefore, haploinsufficiency of *Dotl1* may be compensated by the paternal *Dotl1* gene expression (Liao and Szabó 2020).

#### Histone demethylases

Jumonji and AT rich interactive domain 2 (*JARID2*) mRNA expression is high in both GV and MII bovine oocytes, but its level significantly increased in MII compared with GV oocytes (Fu et al. 2017). Moreover, the expressions of *Kdm1b*, *Kdm1a*, *Kdm5a*, *Kdm5c*, and *Kdm5d* were observed, while *Kdm5b* expression was not present in mouse GV oocytes (Shao et al. 2014). Overexpression of KDM5B, but not KDM5A, led to significantly decreased H3K4me3 and increased transcription level, and this may be an indicative for H3K4me3; KDM5B may be critical for the gene expression process during mouse oocyte maturation (Zhang et al. 2016a). It was shown that *Kdm1a* expression dramatically decreased, while *Kdm2b*, *Kdm4a*, *Kdm6a*, *Kdm6b*, and *Kdm7a* expressions remarkably increased in aged mouse GV oocytes compared with the control (Nishimura et al. 2023). *Kdm2a* expression was the lowest at GVBD stage and at peak at MII stage mouse oocytes. In *Kdm2a* conditional knockout mice, the level of H3K36me2 and H3K36me3 dramatically increased at GVBD stage while number of antral follicle, GVBD oocytes, and maturation rate significantly decreased compared with control. Moreover, *Kdm2a* conditional knockout led to differential expression in 267 genes involved in PI3K-Akt, TGF-β, MAPK, and P53 signaling pathways, which are critical pathways for both folliculogenesis and oogenesis (Xiong et al. 2022). Moreover, the expression level of *JMJD1C* was found to the highest compared with other histone demethyltransferases at both GV and MII stage bovine oocytes (Li et al. 2015). In *Kdm4a*-knockout mouse oocytes, there were no significant differences regarding quality and quantity of oocytes (Sankar et al. 2020). Conditional *Lsd1* (*KDM1A*)-knockout mice revealed chromosomal and spindle abnormality and failed in the resumption of meiosis that ended up with apoptosis during oogenesis. Moreover, it was shown that Lsd1 regulates the activity of CDK1, resulting in meiotic resumption (Kim et al. 2015). Additionally, it is known that aging has a great impact on oocyte competence, and it was demonstrated that the expression of KDM1A increased in aged GV oocytes, while it decreased significantly in aged MII oocytes of mice (Shao et al. 2015). Moreover, KDM1B-deficient mouse oocytes did not alter the proceeding of oogenesis; however, the level of DNA methylation in several imprinting genes, including mesoderm specific transcript (*Mest*), growth factor receptor-bound protein 10 (*Grb10*), and zinc-activated ion channel (*Zac1*) was altered (Ciccone et al. 2009).

### Histone acetylation and deacetylation

In a previous work, we have reviewed histone acetylation during mammalian oogenesis (Bozdemir and Uysal 2023). In a recent study, inhibition of HAT1 in granulosa cells resulted in decreased polar body extrusion rate, increased meiotic defects, and aneuploidy in mouse oocytes (Guo et al. 2023). Moreover, overexpression of KAT8 suppressed superoxide dismutase type 1 *SOD1* gene function, resulting in abnormality in mitochondrial dynamics, redox imbalances, and spindle/chromosome defects during mouse oocyte maturation (Wu et al. 2024). Inhibition of HDAC6 dramatically decreased the oocyte maturation of porcine by increasing the H3K4ac levels (Zhang et al. 2023). In addition, SIN3A, a major scaffolding protein in SIN3–HDAC complexes that participates in histone deacetylation, regulates histone deacetylation during mouse oocyte development (Sun et al. 2023). Furthermore, the inhibition of SIRT5 led to increased histone acetylation and significant meiotic defects, including disrupted spindle organization and chromosome misalignment. In addition, the inhibition of SIRT5 caused mitochondrial dysfunction, subsequently elevating ROS levels and triggering oxidative stress and negatively affecting oocyte maturation (Yan et al. 2025). In another study, it was revealed that HDAC11 levels in follicular granulosa cells and oocytes in Tan sheep increase throughout the growth and maturation of the follicles. In addition, specific inhibition of HDAC11 by SIS17 significantly reduced oocyte maturation rate independently from luteinizing hormone (LH) (Shi et al. 2025). By contrast, the inhibition of HDAC4 in granulosa cells promoted porcine oocyte maturation (Zuo et al. 2025).

### Histone ubiquitination

**Fig. 3:**
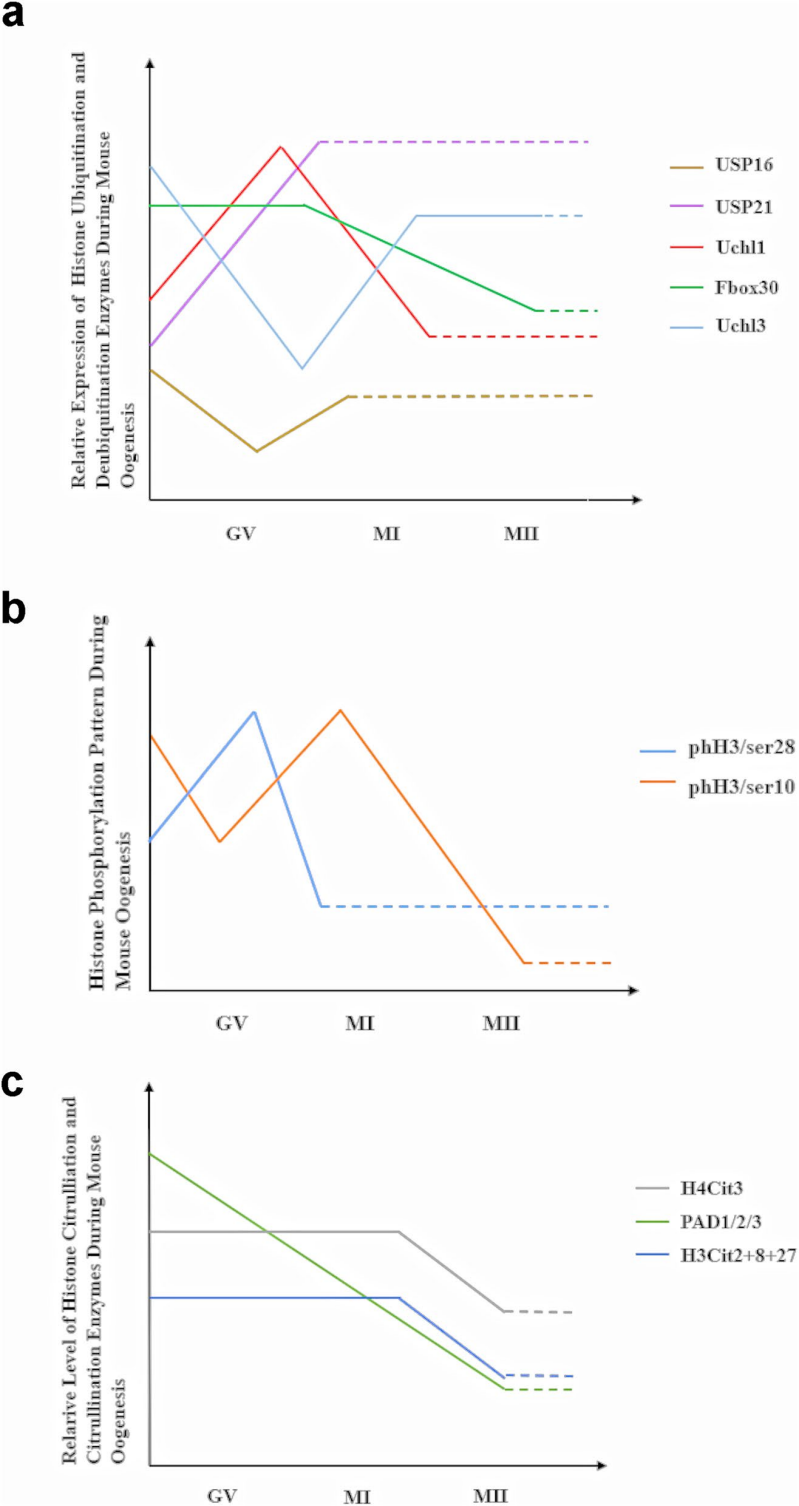
Expression pattern of **a** ubiquitination and deubiquitination enzymes, **b** phosphorylation, and **c** citrullination and responsible enzymes during mouse oogenesis. Dashed line indicates not available. *GV* germinal vesicle, *MI* metaphase I, *MII* metaphase II

S-phase kinase-associated protein 1 (Skp), Cullin, F-box containing complex (SCF) is the largest family in the E3 ligase enzyme group. It contains three domains, which are Skp1, Cullin and Fbox. Fbxo30 is the new member of Fbox family and exists in the mice oocyte during GV to Pre-MI stages and decrease at MI stage. It is indicated that the lowest level of this protein is at MII stage (Fig. 3a). In mice oocytes, Fbxo30 is found at the spindles during pro-MI and MII stages. Depletion of this protein caused significantly compacted chromosomes and abnormal chromosome segregation because of stem-loop-binding protein (SLBP), which is a Fbxo30 substrate. Reduction of Fbxo30 led to a dramatically induced expression of SLBP, and it resulted in excessive H3 accumulation on chromosomes, eventually preventing chromosomal segregation (Jin et al. 2019). In mouse oocytes, ubiquitin carboxy terminal hydrolase L1 (*Uchl1*) expression was found to be low during oogenesis, but it was significantly increased following fertilization. By contrast, *Uchl3* expression was dramatically increased during oocyte maturation. Inhibition of UCHL3 by using ubiquitin-aldehyde (UBAL) and specific inhibitor of UCHL3 separately resulted in abnormality in meiotic spindles and polar body and, eventually, improper oocyte maturation in mouse MII oocytes (Mtango et al. 2012). In mouse and human oocytes, USP16 was the most expressed gene between the genes encoding H2A deubiquitinase. Additionally, the level of *Usp16* was significantly higher in oocytes than other tissues in mice. However, USP16 protein level was not altered during meiosis in mice. In mouse oocytes, the conditional knockout of *Usp16* did not affect cell cycle progression. By contrast, H2AK119ub1 level was not changed in *Usp16* conditional knockout oocytes, while *Usp16* null oocytes revealed a high level of H2AK119ub1 at MII stage (Rong et al. 2022). Moreover, overexpression of USP21 in *Usp26*-null oocytes can decrease the level of H2AK119ub1 (Rong et al. 2022).

### Histone phosphorylation and dephosphorylation

Phosphorylation of H3 on Ser28 (phH3/ser28) and Ser10 (phH3/ser10) during mouse oocyte meiosis was revealed. It was shown that the presence of phH3/ser28 around meiotic chromosomes was persistent during mouse oocyte maturation; however, only in the GV stage, the phH3/ser28 signal was rarely observed on chromatin. By contrast, phH3/ser10 was colocalized with chromatin structure in the GV stage. However, it was observed specifically around centromeric regions at the late anaphase I stage, and it was uniformly distributed along chromosomes at the metaphase II stage (Fig. 3b). Moreover, treatment of oocytes with trichostatin A (TSA), an inhibitor of histone deacetylases (HDACs), resulted in the dephosphorylation pattern of H3/ser10 at centromeric regions during oocyte meiosis and led to abnormal chromosomal segregation (Wang et al. 2006). Moreover, inhibition of phosphatase (PPPs) resulted in hyperphosphorylation of both H3/ser28 and H3/ser10, and it has been revealed that hyperphosphorylation of H3 is required for inducing condensation of chromatin. However, its long term outcome is detrimental, since it led to abnormal chromatin remodeling in mouse oocytes (Swain et al. 2007). Furthermore, p21-activated kinase 1 (Pak1) is responsible for the phosphorylation of H3/ser10, and its inhibition resulted in a significantly decreased proportion of condensed chromosomes. The decreased proportion was improved with treatment of Pak1-activator in mouse oocytes (Zhang et al. 2017). By contrast, in porcine oocytes, decreased H3/ser10 phosphorylation did not cause any abnormality in chromosome condensation (Wang et al. 2013). Additionally, Aurora kinase B is another enzyme responsible for the phosphorylation of Ser10, and its inhibition led to decreased phH3/ser10, but it did not cause an alteration in chromosome condensation in pig oocytes (Jelínková and Kubelka 2006).

### Histone SUMOylation, ribosylation,citrulliation, and lactylation

According to our literature review, there is not any published research demonstrating the presence, absence, or functions of histone SUMOylation during oogenesis in literature. However, there are a few studies about histone citrullination and ribosylation during oogenesis. H4 citrulline 3 (H4Cit3) and H3Cit2 + 8 + 17 were revealed in the nucleus and cytoplasm of mouse GV oocytes; in addition, H3Cit2 + 8 + 17 was shown around meiotic spindles at anaphase II stage (Fig. 3c). Furthermore, it was shown that loss of PAD4 and PAD6 separately did not alter the citrullination level of H4Cit3 and H3Cit2 + 8 + 17 (Kan et al. 2012a). Moreover, PAD1, PAD2, and PAD3 were observed in the nucleus of mouse GV oocytes. Additionally, PAD1 and PAD2 were found on chromosomes, while PAD3 was observed on meiotic spindle in MII oocytes (Zhang et al. 2016c). PAD6 expression has been identified in oocytes (Chavanas et al. 2004). In GV, MI, and MII oocytes, granular citrullinated proteins were shown. Further analysis revealed that histone H3 was a primary target of citrullination in these cells (Brahmajosyula and Miyake 2013). Moreover, Yang et al. investigated the importance of PARP1 in *Parp1*-null mouse oocytes. *Parp1*-knockout female mice were fertile, but the deletion of *Parp1* led to persistent H2AX phosphorylation, chromosomal instability, increased proneness to double-strand breaks (DSBs) (Yang et al. 2009). By contrast, Quian et al. demonstrated the presence of PARylation during folliculogenesis and the presence of PARP1 in the nuclei of oocytes during folliculogenesis until the secondary follicle stage. In addition to that, the inhibition of PARP1 and PARP2 with 5-amninoisoquinolinone (5-AIQ) resulted in significantly increasing in the number of ovulated oocytes and primordial follicles (Qian et al. 2010). In the ovary, lactylation was seen in granulosa cells, stromal cells, and oocytes; the cytoplasm of the oocytes revealed stronger signaling compared with granulosa and stromal cells (Yang et al. 2024). Moreover, immunofluorescence for H3K9la, H3K14la, H4K8la, and H4K12la peaked during the GV stage of mouse oocyte maturation and thereafter diminished or vanished. Additionally, adding 10-mM sodium lactate to the culture medium increased the GV oocytes’ oocyte maturation rate and histone Kla levels, while MII oocytes demonstrated significant increases in Kla levels. It resulted in the alteration in the transcription of genes related with oxidative phosphorylation (Yang et al. 2024). In another study, it was identified that the accumulation of H3K23la, H3K18la, and pan histone lactylation occured in mouse oocytes. Thus, while all these three modifications are absent in GV oocytes, H3K23la and pan histone lactylation was present in the condensed chromosomes of MII oocytes (Yang et al. 2021c)

## Histone modifications during early embryo development

Following ovulation of MII oocytes, in the presence of sperm, gametes fuse and form zygotes. The zygote undergoes mitotic division continuously, and this progresses through the stages of two-cell, four-cell, eight-cell, morula, and blastocyst, respectively. This process is called early embryo or preimplantation embryo development (Li and Winuthayanon 2017). Each cell in the early embryo is called a blastomere. Following eight-cell stages, compaction of blastomeres and their polarization results in two main embryonic cell lineages, inner cell mass (ICM) and trophoectoderm (TE). ICM consists of pluripotent stem cells, and each blastomere in the ICM can form a whole embryo, while trophoblast cells undergo differentiation to form the placenta (Wu and Schöler 2016). Furthermore, preimplantation embryos use maternal mRNAs as templates for protein translation for a certain time before activating their own genome, which is called zygotic genome activation (ZGA), occurring between the 8- and 16-cell stage in human embryos and between the 2- and 4-cell stage in mouse embryos (Sozen et al. 2014). During early embryo development, chromatin undergoes several conformational changes, especially in ZGA; the chromatin conformation is regulated through spatiotemporal presence of posttranslational histone modifications, topologically associated domains (TAD) loop, CCCTC-binding factor (CTCF), and regulatory elements. Thus, formation of higher-order organized chromatin structure is extremely coordinated, and histone modifications are crucial for both gene expression and chromatin structure in early embryo development (Eckersley-Maslin et al. 2018).

### Histone methylation

H3K4me1, H3K4me2, and H3K4me3 dramatically increase in mouse early embryos from the one-cell stage to the two-cell stage, and ZGA occurs between these stages in mouse early embryo development (Fig. 2a. Following ZGA, H3K4me1, H3K4me2, and H3K4me3 decrease significantly until the morula stage. H3K4me1 level is not changed until the blastocyst stage, while H3K4me2 and H3K4me3 remarkably increase at the morula stage and remain at the same level (Shao et al. 2014). Additionally, H3K36me3 is nearly undetectable during mouse and bovine early embryo development; however, the H3K36me3 signal is strongly present in maternal pronuclei of mouse zygote, while it is absent in paternal pronuclei (Bošković et al. 2012). Moreover, in porcine embryos, H3K27me1 demonstrates consistent level until the blastocyst stage, while it significantly increases at the blastocyst stage. H3K27me2 and H3K27me3 dramatically increase at the two-cell stage and significantly decrease at the eight-cell stage, and H3K27me2 markedly increases, while H3K27me3 significantly decreases at the blastocyst stage. However, researchers indicated that the H3K27me3 signal at the blastocyst stage shows heterogeneous pattern. The signal was revealed in only the ICM of 3 embryos, while it was observed in both the ICM and TE of 19 embryos, and no signal was observed in 7 embryos; thus, further investigation is required to understand the histone methylation pattern in ZGA and early embryo development (Marinho et al. 2017). Moreover, recent research demonstrates that maternal factor methyltransferase-like protein 23 (METTL23) catalyzes H3R17me2a, an alteration required for paternal chromatin remodeling and epigenetic reprogramming. This mark promotes the recruitment of the gonad-specific expression–ten-eleven translocation methylcytosine dioxygenase 3 (GSE–TET3) complex and H3.3 histone variant incorporation, which influences the beginning of ZGA. Thus, the accumulation of H3R17me2a, mediated by METTL23, has an indirect influence on ZGA through enabling the required chromatin configuration that promotes histone variant deposition and demethylation (Hatanaka et al. 2017)

#### Histone methytransferases

DOT1L expression is present during mouse early embryo development, but its level dramatically decreases at the two- and four-cell stages and increases at blastocyst stage (Fig. 2b). Decreased expression of DOT1L leads to significantly reduced H3K79me2 in two-cell embryos, while forced expression of DOT1L results in arrest at the two-cell stage (Ooga et al. 2013). By contrast, the inhibition of DOT1L improves the blastocyts rate in porcine somatic cell nuclear transfer (SCNT) and increases expression of the genes’ related lineage differentiation and pluripotency (Tao et al. 2017). *Ash1l* expression reaches its peak at the four-cell stage of porcine embryos (Gao et al. 2010). However, *Ash1l* and *Ash2l* expressions are upregulated at two-cell stage of mouse early embryos, while *Ash1l* maintains a similar expression pattern in the rest of the early embryo stages. *Ash2l* is downregulated between four- and eight-cell stages and upregulated at the morula and blastocyst stage, but not as much as at the two-cell stage (Shao et al. 2014). Additionally, *Ash1l*-mutant female mice are infertile because of an altered uterine environment; however, when they mate with wild-type males and the zygote is transferred into the uterus of a pseudopregnant wild-type female mice, there is no difference between control and *Ash1l*-mutant during embryo development (Brinkmeier et al. 2015). Silencing WD repeat domain 82 (WDR82), which is one of the subunits of SET1A/SET1B complexes, leads to significantly reduced POU5F1 in mouse blastocyst, and it has been shown that downstream proteins of POU5F1 are STAT3 and Birc5, which are crucial in the antiapoptotic pathway. Moreover, knockdown of WDR82 results in a remarkably decreased number of cells in blastocysts due to increased apoptosis, and WDR82-silenced embryos are not viable following embryo transplantation (Bi et al. 2011). By contrast, *Setd1a* expression dramatically decreases at the blastocyst stage compared with previous stages of normal mouse early embryo development (Shao et al. 2014). Therefore, adverse impacts of silencing WDR82 on early embryo development may not be because of its function in SET1A/SET1B complexes. *SETD2* expression in porcine early embryos reaches peak at the eight-cell and morula stages, and knockdown of *SETD2* results in a significant decrease in the cell number of TE; however, it is not effective on the cell number of ICM. In addition, *SETD2* silencing results in the downregulation of genes related to cell lineage differentiation such as *OCT4*, *SOX2*, and *NANOG*, leads to upregulation of proapoptotic gene *BAX* and downregulation of antiapoptotic gene *BCL2*, and eventually leads to an increased apoptotic cell ratio (Shao et al. 2022). Similarly, knockdown of *SETD2* results in dramatically increased DNA damage, apoptosis, and decreased blastocyst rates in mice blastocysts (Li et al. 2020). *Setd7* expression reaches peak at the four-cell stage; then, its level consistently decreases until the blastocyst stage of mouse early embryo development. *Setd8*-null mouse embryos reveals significantly increased early embryonic lethality (Oda et al. 2009; Shikata et al. 2020). *Setdb1 *(*Eset*) expression starts at the blastocyst stage in mouse early embryo development (Cho et al. 2012), and its homozygous deletion results in peri-implantation lethality (Dodge et al. 2004). Furthermore, it is demonstrated that the deletion of maternal *Setdb1* results in severe arrest because of impaired G2/M transition and chromosomal segregation during cleavage stages of mouse early embryo development (Eymery et al. 2016). Additionally, *Kmt2a *(*MLL1*) reaches peak at two-cell stage, while *Kmt2c *(*MLL3*) reaches peak at four-cell stage and *Kmt2b *(*MLL2*) at morula and blastocyst stages (Shao et al. 2014). Moreover, depletion in Mll2 expression results in significantly decreased blastocyst ratio; however, this depletion does not have an impact on cleavage stages of porcine early embryo development (Zhao et al. 2016). Another study highlighted the importance of the H3K4me3 transition, which is mediated by an MLL2-to-SETD1A/B relay mechanism, in regulating the shift from totipotency to pluripotency during the early embryo development of mice (Zhang et al. 2025b). Additionally, Ezh1 expression is absent, while Ezh2 is continuously present during mouse early embryo development (Meng et al. 2020; O'Carroll et al. 2001). Furthermore, *Ezh1* and *Ezh2* double-knockout is not effective on mouse early embryo development, but it results in abnormal embryo development following implantation (Zhao et al. 2022). However, *Ezh2* silencing leads to significantly reduced blastocyst number and increased apoptosis, and it results in a dramatic decrease in the expression of Oct4, Sox2 and Nanog, which are indicators for pluripotency (Huang et al. 2014). Similarly, the heterozygous mutant *Ezh2* blastocyst reveals normal early embryo development, while *Ezh2*-null blastocysts show early embryonic lethality in mice (Erhardt et al. 2003). Moreover, deletion of maternal Ezh1/2 results in severe damage in the morula (Zhang et al. 2024). *Suv39H1* expression reaches peak at the two-cell stage and dramatically decreases at the four-cell stage, and its expression is absent in further bovine early embryo development. By contrast, *Suv39H2* expression is high only in four-cell-stage embryos, and there is significantly lower expression in other stages of early embryo development (Zhang et al. 2016b). *Suv420H1* and *Suv420H2* expressions are the highest at the one-cell stage of mouse embryos, while their expression significantly decreases during early embryo development; additionally *Smyd5* expression reaches peak at the four-cell stage and decreases continuously (Eid et al. 2016). Moreover, *Smyd3* expression reaches peak in four-cell-stage embryos and decreases gradually following this stage during mouse early embryo development. Silencing Smyd3 leads to abnormal gene expression related with lineage differentiation and decreased number of viable offspring (Suzuki et al. 2015). By contrast, Smyd3 expression reaches peak at morula stage. Silencing *Smyd3* in GV oocytes leads to significantly decreased number of eight-cell embryos and impairs further development; however, knockdown of *Smyd3* in a one-cell embryo is not effective on bovine early embryo development (Bai et al. 2016). *PRMT5* expression reaches peak only at 16-cell and blastocyst stages, while *PRMT2* expression is lower at 8- and 16-cell and morula stages than the other stages of bovine early embryos (McGraw et al. 2007). Furthermore, genetic loss of *Prmt1* or *Prmt5* in rodents causes embryonic lethality (Jarrold and Davies 2019). Moreover, Prmt5 homozygous-mutant mice early embryos show upregulation of transposable elements, decreased number of cells, and malformation of blastocoel (Kim et al. 2014). High expression of PRMT7 is revealed in developmentally arrested human early embryos, and PRMT7 silencing results in further embryo development (Zhang et al. 2021). *PRMT4* (*CARM1*) in mouse early embryos is predominantly expressed at the two-cell stage, and PRMT4 silencing leads to significantly decreased blastocyst ratio and increased apoptosis (Zhang et al. 2021). Additionally, the upregulation of *CARM1* results in reduced Cdx2 level, which is a transcription factor leading to TE differentiation in mouse embryos (Parfitt and Zernicka-Goetz 2010). In addition, CARM1 regulated the oviduct epithelial cell exosomes (OEVs) on cell differentiation during in vitro yak early embryo development (Wang et al. 2024). Moreover, in a recent study it was proven that, in mice, the KLF4/PRMT6/H3R2ME2a axis affects trophoblast function, which is involved in unexplained recurrent spontaneous abortion (Tan et al. 2024).

#### Histone demethylases

Deficiency in *KDM1A* causes arrested development at two-cell-stage and fragmented mice early embryos (Ancelin et al. 2016; Wasson et al. 2016). *KDM3A*, *KDM4A*, and *KDM4C* expressions are high at the two-cell stage; following this stage, their levels dramatically decreases throughout bovine early embryo development until blastocyst stage (Pagé-Larivière and Sirard 2014). In addition, *KDM3A* enhances the development of mouse somatic cell nuclear transfer (SCNT) embryos (Matoba et al. 2024). *KDM4A*-null mouse embryos show arrested early embryo development at the two-cell stage and defect in ZGA (Sankar et al. 2020, 2017). The relative expression of *KDM4DL* or its novel isoform *XLOC_004958* is revealed. Their expressions begin at the two-cell stage, and their levels are the highest at the two-cell stage; following the two-cell stage, their expressions consistently decrease throughout early embryo development. Moreover, knockout *KDM4DL* or *XLOC_004958* mouse embryos demonstrate delayed embryo development and significantly increased abnormal blastocyst ratio (Qiao et al. 2020). *KDM6B* knockdown results in decreased blastocyst ratio and decreased cell number of embryoblasts and trophoblasts in bovine embryos (Chung et al. 2017). Furthermore, *KDM7A* silencing results in dramatically increased *KDM3C*, *KDM4B*, and *KDM6B* expression, while it leads to decreased blastocyst ratio and number of cells in porcine parthenogenetic, in vitro fertilized (IVF), and somatic cell nuclear transfer (SCNT) embryos (Rissi et al. 2019). Inhibition of KDM5A and KDM5B leads to significantly increased fragmented embryo ratio in mouse embryos (Dahl et al. 2016). By contrast, the knockdown of KDM5B and KDM5C do not have an impact on cleavage ratio during porcine preimplantation embryo development; however, it causes significantly decreased blastocyst ratio (Glanzner et al. 2020). Moreover, expression of *Kdm1a*, *Kdm1b*, *Kdm5a*, *Kdm5b*, *Kdm5c*, and *Kdm5d* reach peak in four-cell-stage mouse embryos and decrease in further development (Fig. 2c) (Shao et al. 2014). Furthermore, in a recent study, it was shown that LSD1 is essential for mouse embryonic stem cell growth and differentiation by controlling DNA methylation apart from its role in lysine demetylation (Malla et al. 2024). In addition, another research revealed that LSD1 may control autophagy via the Mtor pathway, which affects the early development of porcine embryos (Qi et al. 2024). Additionally, the highest expression of *JMJD1A* is observed at blastocyst stage, while *JMJD2A* expression is downregulated until the eight-cell stage, and, following this stage, its level is upregulated in further stages of bovine early embryo development (McGraw et al. 2007). Moreover, JMJD1C short interfering RNA (siRNA)-treated bovine MII oocytes are fertilized in vitro and cultured in vitro for embryo development until the blastocyst stage, and JMJD1C silencing results in a remarkable decrease in zygote cleavage and blastocyst ratio (Li et al. 2015). *Phf2* expression is at the highest level and decreases consistently throughout mouse early embryo development (Eid et al. 2016). The lowest expression of *JARID1A* is revealed at morula stage, while *JARID1B* expression is absent until the 8-cell stage and reaches peak at the 16-cell and blastocyst stages of bovine early embryo development (McGraw et al. 2007). A study showed that embryos from Jmjd5-deficient mice had significant growth retardation, which led to midgestational death in vivo. The growth deficit was also present in Jmjd5-hypomorphic embryonic fibroblasts from mouse embryos (Ishimura et al. 2012). Moreover, TET3 and primordial germ cell 7 (PGC7) are maternal factors that influence active DNA demethylation in the zygote in an asymmetric manner. TET3 is required for paternal DNA oxidation, whereas PGC7 inhibits this process in the maternal pronucleus through interactions with H3K9me2 (Nakamura et al. 2012)

#### Histone acetylation and deacetylation

In a previous work, we have reviewed histone acetylation during mammalian early embryogenesis (Bozdemir and Uysal 2023). However, within the current study, it is found that H3K27ac regulates ZGA in mice (Zhang et al. 2025a). Moreover, KAT8 controls the transcriptional activity of the trophoblast marker CDX2 by acetylating H4K16. Additionally, deletion of *Kat8* leads to extraembryonic ectoderm abnormalities and embryonic lethality. Moreover, inhibition of SIRT1 by EX527 increased H4K16ac, restored CDX2 levels, and promoted placental development (Bi et al. 2024). Furthermore, fourfold overexpression in *Kat6b* reverses developmental defects in *Kat6a*-mutant mice, restoring hematopoietic stem cells, histone H3K9/23 acetylation, and gene expression (Bergamasco et al. 2025). In addition, a study revealed that selective histone deacetylase inhibitors (HDACi), particularly those targeting Class I HDACs, exhibited stage-specific effects on early mouse embryonic development, with the most pronounced impact observed at the two-cell stage corresponding to ZGA. Treatment with MGCD0103, an inhibitor of HDAC1/2/3, induced a complete developmental arrest at the two-cell stage. By contrast, the HDAC3-specific inhibitor T247 caused a transcriptional delay. Notably, *Hdac7* expression was consistently upregulated in response to both inhibitors, highlighting its potential role in compensatory or regulatory mechanisms following Class I HDAC inhibition (Shao et al. 2024).

### Histone ubiquitination and deubiquitination

The capacity of RING1B, a crucial part of polycomb-repressive complex 1(PRC1), to ubiquitinate H2A is demonstrated to be unnecessary for early mouse embryonic development and a large portion of PRC1’s gene-repression activity (Illingworth et al. 2015). In female trophoblast stem (TS) cells, as well as developing embryonic stem (ES) cells, another polycomb group protein, H2A-K119 ubiquitin E3 ligase Ring1b, is concentrated on the X chromosome (Xi). The enrichment of Ring1b and H2A on the Xi of both TS and ES cells has been shown to be transient during TS and ES cell differentiation, suggesting that Ring1b and H2A are involved in the beginning of both imprinted and random X inactivation. This is consistent with Ring1b mediating H2A ubiquitination (Fang et al. 2004). Researchers found that the H2AK119 deubiquitylation activity of the chromatin modifier USP21 lowers resistance, and they offer proof that H2A ubiquitylation also contributes to resistance to transcriptional reprogramming in mouse nuclear transfer embryos (Jullien et al. 2017). Moreover, according to research, USP22 and ATXN7L3 are necessary for mouse to develop normally during the embryonic stages (Wang et al. 2021). Thus, for cellular ubiquitin equilibrium during mouse embryonic development, H2Bub1 deubiquitination, which is reliant on Spt Ada Gcn5 acetyl-transferase (SAGA) is crucial (El-Saafin et al. 2022). Moreover, mice without Usp16 and Bap1 cannot pass the embryonic stage, whereas mice lacking Usp3, Mysm1, Usp12, and Usp21 are impaired in hematopoietic lineage differentiation, cell cycle control, and DNA repair (Shastry et al. 2024).

### Histone phosphorylation and dephosphorylation

Moreover, ataxia-telangiectasia mutated (ATM) and other protein kinases connected to phosphoinositide three-kinase phosphorylate H2AX. Thus, coupled ATM/H2AX deficit led to significant cellular genomic instability and embryonic mortality in mice (Zha et al. 2008). Furthermore, according to a previous study, exogenous overexpression of Aurora and Ipl1-like midbody-associated protein (AIM-1) in Chinese hamster embryo cells results in increased mitotic Ser-10 phosphorylation with concurrent induction of lagging chromosomes during mitosis. This phosphorylation is necessary for maintaining proper chromosome dynamics during mitosis (Ota et al. 2002). In addition, Haspin is an accepted kinase for H3T3 phosphorylation, and it has been established that it also functions as a downstream kinase for Aurora B in H3.3S31ph in mouse embryonic stem cells (Li et al. 2024b).

### Histone SUMOylation, ribosylation, citrulliation, and lactylation

SUMO1 is present during mouse early embryo development, while SUMO2/3 is undetectable until the four-cell stage, and its level markedly increases in following stages. Inhibition of UBC9, which is an enzyme for SUMOylation, results in abnormal early embryo development, and UBC9-inhibited embryos at the morula stage show a similar transcription pattern to four- and eight-cell-stage embryos (Sheban et al. 2022). Furthermore, PAD1 is observed in nuclei, while PAD2 and PAD4 are present throughout the cytoplasm during mouse early embryo development. By contrast, PAD3 is present in the cytoplasm, except for in the morula and blastocyst stages. Additionally, both specific inhibition and silencing of PAD1 results in significantly reduced H3Cit2/8/17 and H4Cit3, and arrested early embryos at the four-cell stage (Zhang et al. 2016c; Kan et al. 2012b). H4Cit3 and H3Cit2 + 8 + 17 are found in nuclei throughout mouse early embryo development, while H3Cit26 is abundant as clusters on lipid droplets. It is thought that these lipid droplets store histone proteins that may be needed for use during embryonic development (Kan et al. 2012b). PAD4, by contrast, is a transcriptional coregulator that binds to the regulatory regions of important stem cell genes and activates their expression. PAD4 expression and enzymatic activity are also induced during ground state pluripotency and reprogramming. According to previous research, its inhibition lowers the percentage of pluripotent stem cells in the early mouse embryo and significantly reduces reprogramming efficiency (Christophorou et al. 2014). Moreover, PAD6 expression has been identified in embryos (Chavanas et al. 2004). Thus, granular citrullinated proteins have been observed in the nuclei of embryos at all developmental stages, as well as the histone H3, which was a primary target of citrullination in the embryos. Moreover, the colocalization of PAD4 and citrullinated H3 in oocytes and embryos suggests a potential role for PAD4 in preimplantation embryonic development (Brahmajosyula and Miyake 2013). Furthermore, PAD1 catalyzes histone tail citrullination during early embryo development transitions, facilitating early embryo genome activation (Zhang et al. 2016c). According to our literature review, there is not any published research about histone ribosylation in mammalian preimplantation embryos. Moreover, in the mouse zygote, H3K23la, H3K18la, and pan histone lactylation expressions were not dramatic; their expressions increased throughout the cleavage and were remarkable in the blastocyte stage (Yang et al. 2021b). Moreover, throughout the early stages of mouse embryogenesis, histone lactylation levels changed dramatically. While having an effect on the transcription of glycolytic genes, sodium lactate at 10 mM improved early embryo development and markedly enhanced lactylation (Yang et al. 2024). In another research, parthenogenic mouse embryos showed increased ROS levels, and they revealed that adding the walnut-derived peptide TW-7 (Thr-Trp-Leu-Pro-Leu-Pro-Arg) has improved embryo development by facilitating proteins related to histone lactylation such as LDHA, LDHB, and EP300. Thus, the elevated histone lactylation facilitated the expression of ZGA genes (Wei et al. 2024). Another study reported that lactate is present during the ZGA of both human and mouse embryonic development. Inhibition of lactate production leads to major ZGA errors, arrest at the two-cell stage, and loss of H3K18lac (Li et al. 2024a). In line with the previous study, in the absence of sodium lactate, H3K18la reduction occurred and led to arrest at the late G2 phase of the two-cell stage in mouse embryos (Zhao et al. 2024).

## Conclusions

The success of both natural reproduction and assisted reproductive technologies (ART) hinges on the precise control of histone modifications during oogenesis and early embryo development. These modifications exhibit spatiotemporal variation throughout these processes. Since an embryo’s gene expression pattern dictates its fate, these dynamically orchestrated modifications are crucial. Furthermore, the absence or malfunction of enzymes responsible for histone modifications leads to immature oocytes, abnormally developed embryos, infertility, and embryonic death. ART procedures themselves can alter histone modification patterns in embryos, potentially contributing to their lower success rates compared with natural conception. Therefore, a deeper understanding of the precise timing and location of histone modifications during oogenesis and early embryogenesis, along with the underlying mechanisms, could significantly improve ART outcomes. Future research should prioritize a comprehensive analysis of all histone modifications and their interactions with each other during these critical stages.

## Data Availability

No datasets were generated or analyzed during the current study.

## Abbreviations

PMTs: Posttranslational modifications
Lys or K: Lysine
His or H: Histidine
Arg or R: Arginine
PRMTs: Protein arginine methyltransferases
PKMTs: Lysine methyltransferases
SAM: *S*-adenosyl-l-methionine
aDMA: Asymmetric dimethylarginine
MMA: Monomethylarginine
sDMA: Symmetric dimethylarginine
KDMs: Histone lysine demethylases
USP: Ubiquitin-specific proteases
UCH: Ubiquitin C-terminal hydrolases
OTU: Otubain domain ubiquitin-binding proteins
MJD: Machado–Joseph disease protein domain proteases
MINDY: MIU-containing novel DUB family
MCPIP: Monocyte chemotactic protein-induced proteins
ZUFSP: Zinc finger-containing ubiquitin peptidase 1
JAMM: Jab1/MPN domain-associated metalloisopeptidase
SUMO: Small ubiquitin-like modifier
PARP1: Poly (ADP-ribose) polymerase 1
PAD: Peptidyl arginine deiminase
PGCs: Primordial germ cells
GV: Germinal vesicle
GVBD: Germinal vesicle breakdown
MI: Metaphase I
MII: Metaphase II
ICM: Inner cell mass
TE: Trophoectoderm
ZGA: Zygotic genome activation
ART: Assisted reproductive technologies
HAT: Histone acetyltransferase
HDAC: Histone deacetylase
LDHA: Lactate dehydrogenase A
SIRT: Sirtuin
